# Ocular findings and strabismus surgery outcomes in Chinese children with Angelman syndrome: Three case reports

**DOI:** 10.1097/MD.0000000000018077

**Published:** 2019-12-20

**Authors:** Haiyun Ye, Xiaoping Lan, Qingyu Liu, Yidan Zhang, Siying Wang, Ce Zheng, Yue Di, Tong Qiao

**Affiliations:** a Department of Ophthalmology; b Department of Clinical Laboratory, Shanghai Children's Hospital, Shanghai Jiao Tong University, Shanghai, China.

**Keywords:** Angelman syndrome, exotropic, maternal heritage deletion, strabismus surgery

## Abstract

**Rationale:**

Angelman syndrome (AS) is an uncommon genetic disease characterized as serious retarded mental development and ocular abnormality.

**Patient concerns:**

This report aims to present the ophthalmological features, and identify the diagnosis and outcomes of strabismus surgery in AS patients.

**Diagnosis:**

Three children with exotropia were diagnosed with AS based on their typical clinical features.

**Interventions:**

All patients underwent multiplex ligation-dependent probe amplification (MLPA) analysis and accepted lateral rectus recession surgery with the assistance of intravenous combined inhalation anesthesia.

**Outcomes:**

The maternal heritage deletion of chromosome 15q11.2-q13 was verified in all patients by MLPA. All patients with strabismus could not cooperate during the vision test, and had astigmatism. The strabismus type of AS patients was horizontal exotropia, and no vertical strabismus was found. One of these patients was combined with high myopia. The hypopigmentation on the hair and iris was ubiquitous. However, retina pigmentation was normal. After different degrees of lateral rectus recession, the exotropia was significantly relieved, and the surgical effects were stable postoperatively.

**Lessons:**

Horizontal exotropia is the major strabismus type. Severe intellectual disability, hyperactivity, and speech impairment are the common characteristics of AS children. Its examination and operation design remains challenging. Thus, repeated examinations and intelligence rehabilitation are essential.

## 1 Introduction

Dr. Harry Angelman was the first to describe the Angelman syndrome (AS) in 1965. AS is a kind of neurological development-impacting genetic disease.^[1]^ The prevalence of AS has been estimated to be approximately 1 in 15,000 individuals.^[2]^ Furthermore, AS has been verified to be a maternally inherited disorder caused by the dysfunction of the ubiquitin-protein ligase *E3A (UBE3A)* gene that encodes ubiquitin-protein ligase E3A. To date, 4 molecular variants have been identified to be involved in AS pathogenesis: the maternal heritage deletion of the chromosome 15q11.2-q13 critical region, paternal uniparental disomy 15 (UPD), imprinting center defect, and the mutation maternal inherited by the *UBE3A* gene.^[3]^

The consistent characteristics of AS include developmental retardation at approximately 6 to 24 months old, severely impaired ability of language expression, atremble limb movement, ataxic gait, typical signs of microcephaly, and puppet look with a happy personality (frequent laughter and smiling), and ataxia.^[2]^ For the clinical diagnosis of AS, the common accepted criteria is ophthalmic alterations, such as strabismus, iris, and choroidal hypopigmentation, which could be observed in fewer than 80% of affected individuals.^[2]^ During the last few years, many Chinese children with AS were found in our hospital. In the present study, the clinical characteristics of these children, including ocular abnormalities and strabismus surgery outcomes, were summarized. Furthermore, the corresponding literatures were also reviewed.

## 2 Methods

Three children (2 boys and 1 girl) with exotropia were diagnosed with AS based on their clinical futures: poor ability of verbal expression, a wide-based gait, spontaneous laughter, overactive behavior, excitability, and short attention span. The age of these patients ranged from 31 months old to 9 years old. All clinical information of AS children with strabismus was retrospectively reviewed, and parental consent for the publication of this case report was obtained. The deletion testing on the chromosome at 15q11AS-related regions was performed by multiplex ligation-dependent probe amplification (MLPA) with the genetic DNA extracted from the blood of the patients and their parents. Focus was given on the clinical features of the ocular findings and ocular motility. All AS patients underwent lateral rectus recession surgery with the assistance of intravenous combined inhalation anesthesia.

## 3 Case reports

### 3.1 Case 1

**Figure 1:**
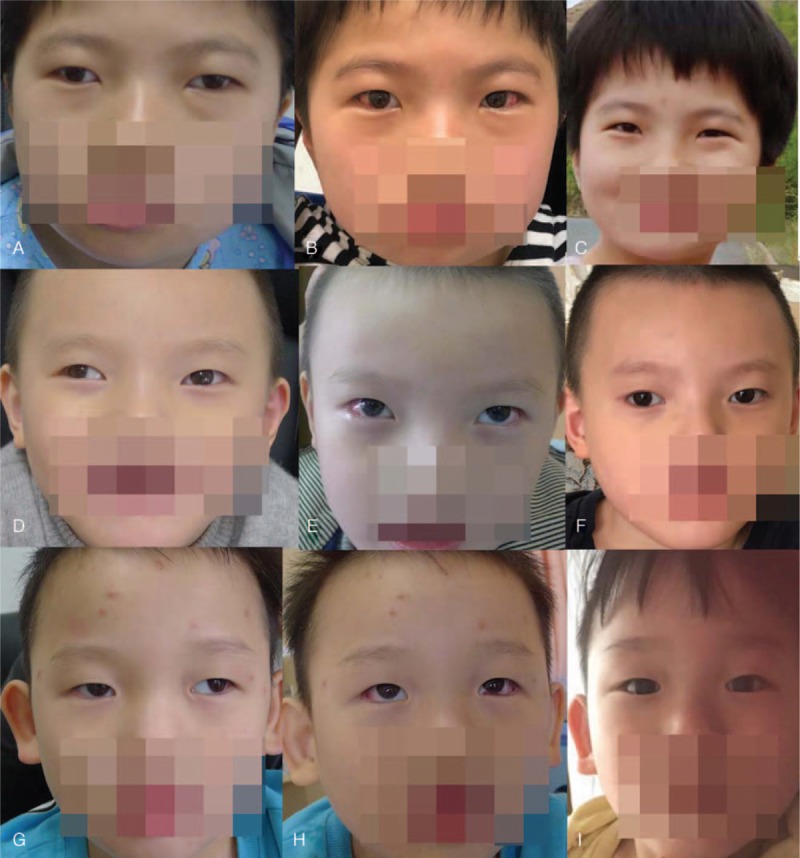
Strabismus surgery outcomes of the 3 AS cases with exotropia. Three AS children (case 1 in A [50Δ], case 2 in D [50Δ], and case 3 in G [60Δ]) presented with exotropia deviation, preoperatively. B, E, and H present the outcomes of the corresponding patients (cases 1, 2, and 3) after 1 d of lateral rectus recession surgery. C, F, and I present the outcomes of the corresponding patients (cases 1, 2, and 3) after 6-mo of lateral rectus recession surgery. Δ: prism diopters.

A 9-year-old girl was admitted to Shanghai Children's Hospital for a happy demeanor, exotropia, and mental retardation. The diagnosis of AS was supported by the genetic DNA examination of the blood sample. The patient had astigmatism combined with high myopia, and the patient's cycloplegic refraction was −12.00/−4.50∗170 in the right eye and −12.00/−4.50∗180 in the left eye. The exotropia deviation of the patient was 50 prism diopters (PD, Fig. 1A). The patient accepted 7.5 mm of lateral rectus recession surgery to repair the strabismus. The patient's exotropia was significantly relieved on the secondary day of surgery (Fig. 1B), and completely returned to normal after 6-months of surgery (Fig. 1C).

### 3.2 Case 2

**Figure 2:**
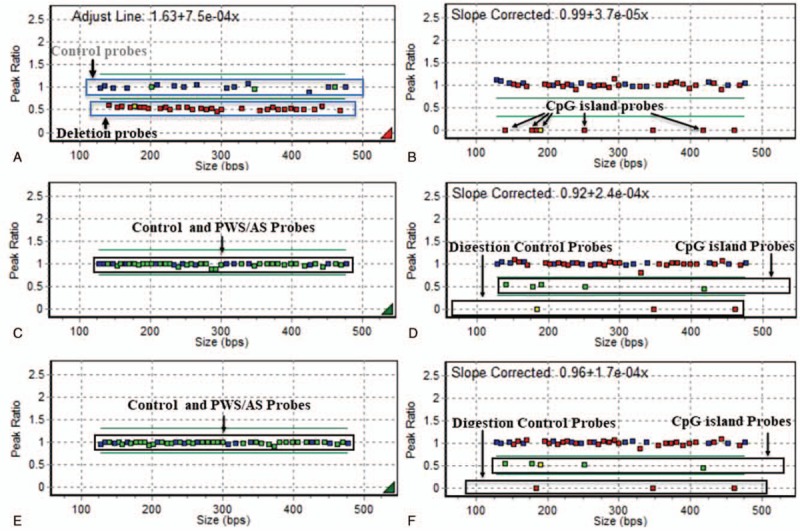
The MLPA analysis of AS child case 2. The genetic DNAs extracted from the blood samples of case 2 (corresponding to Figs. 1D–F) and the child's parents underwent MLPA analysis to detect the heterozygous deletion of *SNRPN, TUBGCP5, UBE3A, MKRN3, GABRB3, ATP10A, MAGEL2, NDN*, and *NIPA* genes in the PWS/AS related regions of chromosome 15q11. The data of A and B reveals the MLPA results of case 2, C and D presents the results of the mother of case 2, and E and F presents the results of the father of case 2. Low methylation in the *SNRPN-in01a, SNRPN-E1, SNRPN-in01b, SNRPN-promoter*, and *NDNa* area was observed, as indicated by the box in the case 2 patient (A and B) and the mother (C and D), indicating that case 2 belonged to the maternal origin deletion. AS = Angelman syndrome, MLPA = multiplex ligation-dependent probe amplification.

Case 2 was a 4-year-old boy, who was referred to our hospital for exotropia and was diagnosed with AS in the Pediatric Genetics Department. The cycloplegic refraction of the patient was −1.00DS/−2.75DC∗10 in the right eye and +0.50DS/−2.75DC∗2 in the left eye. The patient's exotropia deviation was 50 PD, and this patient accepted 7.5 mm of lateral rectus recession surgery to repair the strabismus (Fig. 1D). The heterozygous deletion of the *SNRPN, TUBGCP5, UBE3A, MKRN3, GABRB3, ATP10A, MAGEL2, NDN*, and *NIPA* genes in the Prader-Willi syndrome (PWS)/AS-related regions of chromosome 15q11 was analyzed by MLPA from the genetic DNAs extracted from the blood sample of the patient and the patient's parents. All results indicated that the patient belonged to the maternal origin-15q11AS-related regions of deletion (Fig. 2). The exotropia deviation was well-corrected during the 6-month follow-up period.

### 3.3 Case 3

Case 3 was a 3-year-old boy with exotropia and co-existent trichiasis. The patient was diagnosed with AS by MLPA. The cycloplegic refraction of the patient was +2.00/+2.00∗100 in the right eye and +2.00/+2.00∗85 in the left eye. The patient's exotropia deviation was 60 PD. The patient accepted 8 mm of lateral rectus recession surgery (Fig. 1H), and revealed effective outcomes during the follow-up period (Fig. 1I).

## 4 Discussion and review

The majority of AS cases are sporadic. Nevertheless, several reports have shown that AS may recur with familial transmission.^[4]^ The present data shows that the patients in the present study belong to sporadic AS. AS has characteristics of pleasant expression and severe developmental delay.^[5]^ Three patients had severe developmental delay at approximately 2 to 4 years old. However, none of these patients could cooperate during the vision test due to their strabismus. Furthermore, the clinical representation of AS was highly inconstant, which would have overlapped with the Angelman-like syndrome, including Rett syndrome, Kleefstra syndrome, Mowat-Wilson syndrome, and FOXG1-related disorder.^[6]^ The diagnosis of AS was based on typical clinical manifestations and/or genetic DNA sequence analysis.^[7]^ However, genetic testing results could exhibit a deletion of maternal material or uniparental disomy of paternal material, which may distinguish AS from these. Thus, geneticists recommend that a methylation study of the 15q11-13 region should be performed first for suspected AS patients, and subsequently analyze the *UBE3A* gene when the study was negative.^[8]^ In the present study, the methylation on the chromosome 15q11-13 region was confirmed in 3 children and their mothers, indicating that they had a deletion defect on the 15q11-13 region. Thus, the *UBE3A* gene analysis was not preceded. All patients in the present study were diagnosed based on both typical clinic futures and genetic analysis.

### 4.1 Ocular motility and pigmentation

Michieletto et al reported that the prevalence of strabismus was correlated with genetic causes. Furthermore, the incidence rate of strabismus was 100% in the genetic mutation group. Although the data was not specific, exotropia is the most frequent style of deviation error, according to a literature.^[9]^ The strabismus type of the AS patients in the present series was mainly horizontal exotropia, and no vertical strabismus was observed at present. The exotropia deviation of these present patients ranged within 50 to 60 PD (A, D, and G). All patients accepted different degrees of lateral rectus recession surgery to repair their strabismus, and obtained effective outcomes. Their exotropia was significantly relieved on the secondary day of surgery (B, E, and H), and completely returned to normal after 6-months of surgery (Fig. 1C, F, and I). All patients were followed-up from 6 months to 2 years, revealing that the postoperative effects were stable.

Hypopigmentation could entirely or only affect the ocular, hair, or skin. Ocular hypopigmentation is divided into 4 phases, and applied to determine the ocular phenotype in patients with albinism. Autosomal recessive genetic mutation at the *P* gene is the etiological cause of the most common tyrosinase-positive albinism-oculocutaneous albinism type 2.^[9,10]^ Michieletto et al considered that at least 1 other mutated gene deleted locus at 15q11.2-q13 alone or jointly with the *P* gene should be responsible for the pigmentation.^[9]^ In the present report, the pigmentation of the skin, hair, retina, and iris of these patients was compared with those of their parents. Interestingly, all 3 Chinese children with AS only presented with hypopigmentation on the skin, hair, and iris, and not on the retina.

### 4.2 Refraction

Notably, the Scientific Advisory Committee of the United States Angelman Syndrome Foundation does not list refractive error in the AS frequent clinical feature at present.^[9,11]^ Keratoconus has been suggested to be a common symptom.^[9]^ The refractive symptom from present AS cases revealed that the refractive errors were ubiquitous, and astigmatism was the most frequently observed sign, which is in coincidence with previous reports.^[9]^ Likewise, 1 patient in the present study (case 1) had high myopia. Consistent with many studies, astigmatism is present with very high frequency. Therefore, a number of ophthalmologists have suggested that refractive error must to be considered as a frequent feature of AS.^[9]^ The present data supports this opinion.

### 4.3 Anesthetics and surgery

Strabismus commonly occurs in stunted children, but its surgical results of exotropic AS had rarely been published, to date. Due to the common comorbidity of severe intellectual disability, hyperactivity, and speech impairment in AS children, its examination or operation design remains challenging.^[12]^ Thus, repeated observations are necessary. Furthermore, the assistance from their parents would be helpful for preoperative consultation.

In the present study, 3 AS patients received intravenous combined inhalation anesthesia during surgery. These AS patients generally tolerated the anesthetic management without additional sleep disturbances after the operation. However, Warner et al indicated that the speech obstacle or happy demeanor of AS patients could confuse the postoperative pain assessment.^[12]^ In addition, they also suggested that the anesthesiologist should modify the anesthetic management based on the features of AS. Our experience suggests that the indications for surgery are the same as those for a normal exotropia operation. The selection of lateral rectus weakening surgery is the major strategy. Bilateral lateral rectus recession with the surgical doses is the same as a standard exotropia surgery. All patients were followed-up from 6 months to 2 years, revealing that the postoperative effects were stable.

### 4.4 Genetic mutation of AS

Although the diagnostic criteria of the electroencephalogram examination were considered to be predominant for the diagnosis of AS, genetic analysis remains fundamental for the final diagnosis of AS.^[13,14]^ It has been well-accepted that the dysfunction of maternally inherited *UBE3A* is caused by 4 different mechanisms. The deletion at the maternal chromosomal region of 15q11-q13 is the most frequent etiology. Indeed, all patients in the present study falls into this category^[5,15,16]^ (Fig. 2). AS patients with a gene deletion had more severe phenotypes, when compared to patients with only UPD or imprinting defects.^[5,15]^

The diagnostic algorithm begins with the determination of chromosome 15q11-q13 DNA methylation. Further etiological testing can determine whether any DNA code deletion, an imprinting flaw, or UPD exists.^[6]^ Fluorescence in situ hybridization and chromosomal microarray can determine the deletion and refine the deletion size. The deleted range has been reported to correlate with the clinical features of severity. UPD can be verified or excluded through the DNA marker analysis of the chromosome 15q11-q13 region. When both the 15q11-q13 deletion and UPD are excluded by abnormal DNA methylation, it can be considered that the AS patient has an imprinting defect.^[6]^ In conclusion, the ocular findings and strabismus surgery outcomes in 3 children with exotropic AS were observed. Furthermore, the literature on AS anesthetics and genetic diagnosis was also reviewed. However, due to the low incidence rate of AS, the present number of patients was limited. More research is needed in the future to explore the outcomes of AS strabismus. AS individuals need care through their full lifespan. However, no effective therapeutic intervention presently exists. In addition to the strabismus surgery, these cases also need measures to improve the intelligence and rehabilitation nursing. Based on the above opinions, it is suggested that the termination of pregnancy for mothers with an AS fetus through the genetic analysis of amniotic fluid remains as the most effective precaution.

## Author contributions

**Data curation:** Tong Qiao, Xiaoping Lan.

**Formal analysis:** Haiyun Ye, Qingyu Liu.

**Investigation:** Haiyun Ye, Yidan Zhang, Yue Di.

**Methodology:** Tong Qiao, Haiyun Ye.

**Resources:** Tong Qiao, Siying Wang.

**Validation:** Xiaoping Lan.

**Writing – original draft:** Haiyun Ye.

**Writing – review and editing:** Ce Zheng, Tong Qiao.
